# Supplementary material for: Cystinosin is involved in Na+/H+ Exchanger 3 trafficking in the proximal tubular cells: new insights in the renal Fanconi syndrome in cystinosis
Source: bioRxiv. 2025 Feb 12:2025.02.12.637793. Preprint. [Version 1] doi: 10.1101/2025.02.12.637793 (PMC11844504; doi:10.1101/2025.02.12.637793)
Supplement: 1 [file NIHPP2025.02.12.637793V1-supplement-1.pdf]

bioRxiv preprint doi: https://doi.org/10.1101/2025.02.12.637793; this version posted February 12, 2025. The copyright holder for this preprint (which was not certified by peer review) is the author/funder, who has granted bioRxiv a license to display the preprint in perpetuity. It is made available under aCC-BY 4.0 International license.

Figure S1. - Sequence alignments and AlphaFold-predicted structures of cystinosin protein isoforms in humans, *P. pastoris*, and *S. cerevisiae*. (A) Clustal Omega multiple sequence alignment of human cystinosin (Uniprot accession number A0A0S2Z3K3), cystinosinLKG (A0A0S2Z3I9), *P. pastoris* Ers1S (C4R5N7), *P. pastoris* Ers1L (C4R120), and *S. cerevisiae* Ers1 (P1761). Black boxes indicate amino acid identity, grey boxes indicate amino acid similarity, with the red-boxed area highlighting the comparison of the seven-transmembrane domains of cystinosin across the different organisms. We used BoxShade for highlighting (https://junli.netlify.app/apps/boxshade/#forms::boxshade). The multiple sequence alignment was performed using Clustal Omega from EMBL (https://www.ebi.ac.uk/jdispatcher/msa/clustalo). The threshold for comparison is set by default to greater than 50%. **(B)** Displays images of the AlphaFold-predicted structures of the various cystinosin isoforms in each organism described in **(A).**

Figure S2. - Cystinosin does not interact with NHE1. Immunoprecipitation assay performed in HEK293T cells stably expressing NHE1, cystinosin, and cystinosinLKG showing no interaction between NHE1 and both cystinosin and cystinosinLKG. Input lysates show proper expression of the proteins with glyceraldehyde-3-phosphate dehydrogenase (GAPDH) used as a loading control. Pull down with anti-IgG was used as a negative control.

Figure S3. - Cystinosin deficiency impairs NHE3 trafficking in human WT and *CTNS*$^{-/-}$ HK2 proximal tubular cells. (**A & B)** Representative immunofluorescence images of NHE3-GFP (green) with markers of lysosome (LAMP1) (red) (**A**) and Golgi (GM130) (red) (**B**) with their corresponding quantification showing defect in NHE3 subcellular localization in *CTNS*$^{-/-}$ HK2 cells. Scale bars = 20 μm Bar graphs are presented as the mean $\pm$ SEM. *$P < 0.05$; ***$P < 0.001$ using two-tailed Student's t test.

bioRxiv preprint doi: https://doi.org/10.1101/2025.02.12.637793; this version posted February 12, 2025. The copyright holder for this preprint (which was not certified by peer review) is the author/funder, who has granted bioRxiv a license to display the preprint in perpetuity. It is made available under aCC-BY 4.0 International license.

Figure S4. GFP$^+$ WT HSPC distribution in the kidney following WT HSPC transplantation. Overview of immunofluorescence images of entire kidney section from the Test mice stained with anti-GFP (green), anti-NHE3 (magenta) and anti-LTL (red) antibodies. This image shows the distribution of the GFP$^+$ WT HSPC-derived cells as well as the colocalization of LTL and NHE3 within the kidney of *Ctns*$^{-/-}$ mice transplanted with WT HSPCs. Scale bars = 500µm.

Supplementary Tables

Table S1. *Pichia pastoris* strains

Description	Strain	Genotype	Reference
WT	PPY12	*his4, arg4*	1
WT + Sec7-DsRed	PPY12	PPY12 Sec7-DsRed*::SEC7*(*HIS4* selection) *arg4 his4*	2
Δ*ers1S*	Sjcf1545	PPY12 *Δers1S::ZEOCIN arg4 his4*	This study
Δ*ers1L*	Sjcf1519	PPY12 *Δers1L::KanMX arg4 his4*	This study
Δ*ers1S* Δ*ers1L*	Sjcf1546	PPY12 *Δers1S::ZEOCIN Δers1L::KanMX arg4 his4*	This study
Δypt7	Srrm197	PPY12 *Δypt7::KanMX arg4 his4*	3
Δvps1	Sjcf1614	PPY12 *Δvps1::ZEOCIN arg4 his4*	This study
Δvps15	OP5	GS200 *Δvps15::ScARG4 arg4 his4*	4

Table S2. *Pichia pastoris* plasmids

Plasmid	Promoter	Fusion Protein	Integration Locus	Selectable Marker
pJCF728	*ERS1S*	Ers1S-GFP+	*ARG4*	*ARG4*
pJCF155	*NHX1*	Nhx1-GFP	*ARG4*	*ARG4*
pTA12	*VPS8*	Vps8-2xmCherry	*VPS8*	*NAT*
pTA8	*NHX1 / ERS1S*	Nhx1-VC / Ers1S-VN	*ARG4*	*ARG4*
pTA3	*NHX1*	Nhx1-VC	*HIS4*	*HIS4*
pTA2	*ERS1S*	Ers1S VN	*HIS4*	*HIS4*

References

1 Gould, S. J., McCollum, D., Spong, A. P., Heyman, J. A. & Subramani, S. Development of the yeast Pichia pastoris as a model organism for a genetic and molecular analysis of peroxisome assembly. *Yeast* **8**, 613-628 (1992). https://doi.org:10.1002/yea.320080805

2 Soderholm, J. *et al.* The transitional ER localization mechanism of Pichia pastoris Sec12. *Dev Cell* **6**, 649-659 (2004). https://doi.org:10.1016/s1534-5807(04)00129-7

3 Manjithaya, R., Anjard, C., Loomis, W. F. & Subramani, S. Unconventional secretion of Pichia pastoris Acb1 is dependent on GRASP protein, peroxisomal functions, and autophagosome formation. *J Cell Biol* **188**, 537-546 (2010). https://doi.org:10.1083/jcb.200911149

bioRxiv preprint doi: https://doi.org/10.1101/2025.02.12.637793; this version posted February 12, 2025. The copyright holder for this preprint (which was not certified by peer review) is the author/funder, who has granted bioRxiv a license to display the preprint in perpetuity. It is made available under aCC-BY 4.0 International license.

4 Stasyk, O. V. *et al.* A Pichia pastoris VPS15 homologue is required in selective peroxisome autophagy. *Curr Genet* **36**, 262-269 (1999). https://doi.org:10.1007/s002940050499

Table S3. Primer sequences for qPCR. All primers were reconstituted at 100 µM and used at a working concentration of 5 µM.

**Gene**	**Full Name**	**Purpose**	**Direction** (5'-3')	**Sequence**
**Human Primers**				
GAPDH	**glyceraldehyde-3-phosphate dehydrogenase**	qPCR Housekeeping	Forward<br>Reverse	TCAAGGCTGAGAACGGGAAG<br>CGCCCCACTTGATTTTGGAG
CTNS	Cystinosin	mRNA Expression	Forward<br>Reverse	TCCTCCTGTCGTAAAGCTGGA<br>GCCGGTCTGATTGGAGTGAT
NHE3	Na+/H+ exchanger 3	mRNA Expression	Forward<br>Reverse	GTCACCGTGGTTCTGTACAAT<br>CACCACGAAGAAGGACACTATG
NHE2	Na+/H+ exchanger 2	mRNA Expression	Forward<br>Reverse	TCAAATCCGTCAGCGAACTTT<br>TTCTCGCAAACTGTGTCGCC
NHE1	Na+/H+ exchanger 1	mRNA Expression	Forward<br>Reverse	CACTGTCATCTTCTTCACCGTC<br>ATCTCTTCGTTGATGGAGCG
**Murine Primers**				
Gapdh	glyceraldehyde-3-phosphate dehydrogenase	qPCR Housekeeping	Forward<br>Reverse	GCACAGTCAAGGCCGAGAAT<br>GCCTTCTCCATGGTGGTGAA

bioRxiv preprint doi: https://doi.org/10.1101/2025.02.12.637793; this version posted February 12, 2025. The copyright holder for this preprint (which was not certified by peer review) is the author/funder, who has granted bioRxiv a license to display the preprint in perpetuity. It is made available under aCC-BY 4.0 International license.

Ctns	Cystinosin	mRNA Expression	Forward<br>Reverse	CCACATGGCTCCAGTTCCTCT<br>CACACCGCCAATGCTCCAG
Nhe3	Na+/H+ exchanger 3	mRNA Expression	Forward<br>Reverse	ATGCAGTGACTGTGGTCTTG<br>CACCACGAAGAAGGACACTATG
Nhe2	Na+/H+ exchanger 2	mRNA Expression	Forward<br>Reverse	ATAAGGAAGCTCACGCCAG<br>CCGTCAGGTTGTGTCTGTTAT
Ctns WT	Cystinosin	Genotyping	Forward<br>Reverse	CTCCAGATGTTCCTCCAGTC<br>AGTCCGAACTTGGTTGGGT
Ctns mutant	Cystinosin	Genotyping	Forward<br>Reverse	GCAGGAATTCGATATCAAGC<br>AAAGTGGAGGTAGGAAAGAGG

Table S4. Donor-derived HSPC engraftment in *Ctns*$^{-/-}$ mice transplanted with GFP^{+} HSPCs

Mice (*Ctns*$^{-/-}$)	Gender	Engraftment of GFP^{+} cells in blood (%)
1.	Female	76.5
2.	Male	69.7
3.	Male	73
4.	Female	77.2
5.	Female	6.9
6.	Male	57.6
7.	Male	57.3
8.	Female	73.7
9.	Male	70.7
10.	Male	49.3

bioRxiv preprint doi: https://doi.org/10.1101/2025.02.12.637793; this version posted February 12, 2025. The copyright holder for this preprint (which was not certified by peer review) is the author/funder, who has granted bioRxiv a license to display the preprint in perpetuity. It is made available under aCC-BY 4.0 International license.

SUPPLEMENTARY METHODS

Lentiviral particle production

HEK293T cells line was cultured in DMEM supplemented with 10% heat inactivated FBS, with a prescribed dose of Penicillin/Streptomycin (Invitrogen, Life Technologies) added as recommended antibiotics. All cells were maintained at 37°C in a humid incubator with 5% CO2. Lentiviral particles were produced using the 3 packaging plasmids (pMDLg/pRRE, pHCMV-g, pRSV-Rev) (12) along with the plasmid carrying *CTNS-DsRed*, *CTNS-LKG-DsRed*, and *NHE1-GFP*, *NHE2-GFP*, *NHE3-GFP* and were transfected using calcium phosphate protocol (12). Vector particles were harvested in media and concentrated through ultracentrifugation at 25,000 rpm for 2 hours at 4°C. The titers of the concentrated virus, finally dissolved in Stemspan medium (StemCell Technologies, Vancouver, British Columbia, Canada), were determined by infecting HEK293T cells with serial dilutions of the virus preparations and evaluating them via flow cytometry and droplet digital (dd) PCR. Subsequently, the virus was introduced to HEK293T and maintained for 14 days to establish the stable cell lines. The GFP and DsRed positive cells were selected using fluorescence-activated cell sorting (FACS).

Yeast studies

Strains and plasmids used are shown in Supplementary Table S1 and S2, respectively. Medium used in this study: YPD (2% glucose, 2% bacto-peptone, 1% yeast extract), YNB (0.17% yeast nitrogen base without amino acids and 0.5% ammonium sulfate), YNB-N (0.17% yeast nitrogen base without amino acids and ammonium sulfate), CSM (complete synthetic medium of amino acids and supplements), glucose medium (2x YNB, 0.79g/L CSM, 0.04 mg/L biotin, 2% dextrose), SD-N or starvation medium (1x YNB-N and 2% dextrose)

Extraction of intracellular cystine. Samples (50 ml) of cultures were harvested by centrifugation at 2,000 g and washed twice in 100 mM phosphate buffer (pH 7.5). The cells were suspended in 5% trichloroacetic acid (TCA) in water and broken with 0.5-mm glass beads. Cell debris was removed by centrifugation (10,000 g) at 4°C, and the supernatant was stored at -20°C for MS analysis of cystine content.

bioRxiv preprint doi: https://doi.org/10.1101/2025.02.12.637793; this version posted February 12, 2025. The copyright holder for this preprint (which was not certified by peer review) is the author/funder, who has granted bioRxiv a license to display the preprint in perpetuity. It is made available under aCC-BY 4.0 International license.

Fluorescence Microscopy. Cells were grown in YPD at 30°C until exponential phase (1–2 OD600/mL), washed twice with sterile water, and then transferred to glucose medium for 6 hours. Mid-log cells were then pelleted, 1.5 µl of cells was mixed with 1% low melting point agarose and placed on a glass slide with a coverslip and imaged using 63× or 100× magnification on a Carl Zeiss Axioskop 2 MOT microscope (Carl Zeiss Microscopy, Gottingen, Germany). Images were taken on an AxioCam HRm digital camera (Carl Zeiss MicroImaging GmbH, Gottingen, Germany); no digital gain was used, exposure was adjusted as needed, except for BiFC which was kept constant during microscopy in different strains. Images were processed using AxioVision software V4.8.2.0 (Carl Zeiss Microscopy, White Plains, NY, USA). The images are representative results from experiments conducted at least in triplicate. Methodology to determine late endosome (LE) or trans-Golgi network/early endosome (TGN/EE) localization in different background strains is as follows: Cells showing distinct LE labeling with Vps8-2xmCherry or TGN/EE labeling with Sec7-DsRed were first marked in the red channel in the AxioVision software. Then, these marks were analyzed for colocalization in the green channel with the GFP-tagged proteins or fluorescence obtained from Bimolecular Fluorescence Complementation **(**BiFC). FM4-64 staining was visualized after a short pulse (~3 min) followed by a chase in the presence of the quencher (4-Sulfonato calix [8] arene, sodium salt) SCAS (BIOTIUM cat#70037).

Immunoprecipitation (IP) assay

HEK293T stably transduced with various combinations of LV-CTNS-DsRed; LV-CTNS-LKG-DsRed, LV-NHE1-GFP, LV-NHE2-GFP, and LV-NHE3-GFP, were cultured and harvested from 15 cm dish plates. Whole cell extracts were prepared using IP Lysis Buffer (50 mM Tris-HCL pH 7.5, 150 mM NaCl, 1mM EDTA, 1% Igepal CA-630, 10% Glycerol, 0.5 mM DTT) supplemented with freshly added PIC, PMSF & DTT. Supernatants were collected by centrifugation at 13000 x g for 10 min at 4°C. Protein conc. was measured by Pierce BCA Protein Assay Kit. 10% Input was kept at -80°C for later use. Prior to immunoprecipitation (IP), lysates underwent pre-clearing via incubation with Dynabeads at 4°C for 30 mins. GFP pull-down was executed using GFP-Trap Magnetic Beads (Chromotek-gtma), while DsRed pull-down utilized Protein A Dynabeads pre-coupled with RFP Antibody (Chromotek-6G6) at 4°C for 2-4 hrs. The pre-coupled beads were then incubated with the 500ug-1mg protein lysate at 4°C O/N. Next day, the tubes were replaced,

bioRxiv preprint doi: https://doi.org/10.1101/2025.02.12.637793; this version posted February 12, 2025. The copyright holder for this preprint (which was not certified by peer review) is the author/funder, who has granted bioRxiv a license to display the preprint in perpetuity. It is made available under aCC-BY 4.0 International license.

and the beads were washed with IP Lysis Buffer followed by IP Wash Buffer (50mM Tris-HCL pH 7.5, 200mM NaCl, 1mM EDTA, 1% Igepal CA-630, 10% Glycerol,1mM DTT) (with freshly added PIC, PMSF & DTT). Elution of proteins was achieved using 30 µl of 2X protein loading dye. Co-immunoprecipitated proteins were resolved by SDS-PAGE and probed for GFP (Abcam, ab290) and DsRed (Chromotek-6G6). Pulldown using normal mouse IgG (sc-2025) or normal rabbit IgG (CST-2729) was used as a negative control.

Immunoblotting

Cells and murine kidney tissues were homogenized in Pierce RIPA buffer (Sigma, St Louis, MO) supplemented with Protease Inhibitor Cocktail (Sigma, St Louis, MO). Protein concentrations were determined using the Pierce BCA Protein Assay Kit and 30-50µg of proteins were loaded onto SDS-PAGE gels for subsequent immunoblotting following standard protocols. The proteins transferred onto the PVDF membrane was incubated with the primary antibodies at a 1:1000 dilutions in 5% BSA in TBS-T, with overnight incubation at 4°C for primary antibodies and 1-hour incubation at room temperature for secondary antibodies. The primary antibodies used were rabbit anti-NHE3 (Millipore, AB3085), rabbit anti-NHE2 antibody (Novus, NBP2-38236), mouse anti-NHE1 antibody (Proteintech, 67363-1-1g) and mouse anti-GAPDH antibody (Proteintech, HRP-60004) followed by goat anti-mouse or anti-rabbit horseradish peroxidase-conjugated secondary antibodies. Blots were developed using Enhanced Chemiluminescence (ECL) reagent according to the manufacturer's protocol (GE Healthcare, Pittsburgh, USA) and imaged using the Azure 600 imager (Azure Biosystems, Dublin, CA, USA). Quantification of bands was carried out using GelQuant.Net software.

RNA extraction and real-time quantitative PCR (RT-qPCR)

Total RNA was extracted from both cells and mice kidney (after homogenization in Precellys 24) employing the RNeasy Mini Kit as per the manufacturer's protocol (Qiagen, Hilden, Germany, cat. 74104). Subsequently, 500 ng of RNA was transcribed into cDNA using iScript cDNA Synthesis Kit (Bio-Rad, Hercules, CA, cat. 1708840). For the RT-qPCR reaction setup, 5 µl of iTaq Universal SYBR Green Supermix (Bio-Rad, Hercules, CA, cat.1725121), 3 µl of 1:10 diluted cDNA (2.5 ng/µl), and 1 µl of forward and reverse primer (5 µM each) were combined. The reaction was performed on a CFX96 thermocycler (Bio-Rad) under the following conditions: 95°C

bioRxiv preprint doi: https://doi.org/10.1101/2025.02.12.637793; this version posted February 12, 2025. The copyright holder for this preprint (which was not certified by peer review) is the author/funder, who has granted bioRxiv a license to display the preprint in perpetuity. It is made available under aCC-BY 4.0 International license.

(30 s); 40 cycles of 95°C (5 s) and 60°C (30 s); then 65°C (5 s); and 95°C (5 s). Gene expression was quantified utilizing the ΔΔCt method relative to the wild-type (WT) and normalized to the endogenous control (GAPDH). All primer sequences are shown in Table S3.

Determination of Intracellular Sodium

To measure intracellular sodium concentration [Na^+], we used the sodium ion fluorescence indicator Sodium Green™ Tetraacetate (Invitrogen, Catalog #S6901) (92). We seeded 0.6×10^6 cells in 24-well plates overnight. The next day, the cells were washed twice with PBS and incubated with Sodium Green™ Tetraacetate at a final concentration of 2μM in dimethyl sulfoxide for 8 minutes. After incubation, the cells were washed three times with PBS to remove excess dye and processed for flow cytometry (BD Accuri C6, BD Biosciences). AAD-7 was used to exclude dead cells. All flow cytometric analyses were performed using BD software. We also conducted this assay following cells treated with NHE3 inhibitor EIPA (5-(*N*-ethyl-*N*-isopropyl)-amiloride (Sigma) at a concentration of 100 μM for 4 hrs. DMSO was used as the vehicle control for EIPA treatment with a final concentration of 0.1%.

Albumin Uptake Assay

To assess the cells' ability to uptake albumin, 0.6×10^6 cells were seeded in a 24-well plate overnight. The next day, the cells were washed twice with PBS and incubated with 50 μg/mL Alexa Fluor 555-conjugated albumin (Invitrogen, Cat #A34786) for approximately 16 hours for complete intracellular uptake and processing of albumin. After incubation, the cells were washed three times with PBS to remove excess dye and processed for flow cytometry (BD Accuri C6, BD Biosciences). AnnexinV (Invitrogen, Cat #331200) & 7-amino-actinomycin D (7-AAD) (Invitrogen, Cat # 00-6993-50) was used to exclude dead cells. All flow cytometric analyses were conducted using BD software. We also conducted this assay following cells treated with NHE3 inhibitor EIPA (5-(*N*-ethyl-*N*-isopropyl)-amiloride (Sigma) at a concentration of 100 μM for 4 hrs.

Total internal reflection fluorescence microscopy (TIRFM) and data analysis

For live-cell TIRFM imaging, human PTCs were seeded in 8-well plates with coverglass bottoms (Lab-Tek borosilicate, Nunc, Thermo) in phenol red-free RPMI medium. Cells were placed on a prewarmed microscope stage, and imaging was done using a 100× 1.45 numerical aperture (NA)

bioRxiv preprint doi: https://doi.org/10.1101/2025.02.12.637793; this version posted February 12, 2025. The copyright holder for this preprint (which was not certified by peer review) is the author/funder, who has granted bioRxiv a license to display the preprint in perpetuity. It is made available under aCC-BY 4.0 International license.

TIRF objective on a custom-modified Nikon TE2000U microscope with TIRF illumination. Laser illumination (488 and 543 nm) was angled to create an evanescent field depth of <100 nm. Images were captured on a 14-bit cooled CCD camera (Hamamatsu) controlled by NIS-Elements software (Nikon) at 1-second intervals with 200–600 ms exposure times. Analysis was performed using ImageJ (version 1.43) and Imaris (version 7.0, Bitplane Scientific Software). Granule movement was tracked across all movie frames, including vesicles visible in the TIRFM zone for at least three frames. Images with mild fading were auto-thresholded in Imaris to ensure consistent tracking.

Hematopoietic stem and progenitor cell (HSPC) isolation, transplantation, and engraftment

Bone marrow cells were extracted from the femurs of 6- to 8-week-old *Ctns*$^{-/-}$ or WT-GFP^{+} transgenic mice. HSPCs were isolated through immunomagnetic separation utilizing an anti-Sca1 antibody linked to magnetic beads (Miltenyi Biotec). The ~2 × 10^6 Sca1^{+} HSPCs cells suspended in 100 uL of phosphate-buffered saline (PBS) were then directly injected via tail vein into previous day lethally irradiated (7 Gy; X-Rad 320, PXi) *Ctns*$^{-/-}$ mice. In the case of mice receiving WT GFP^{+} HSPCs, engraftment of the transplanted cells was assessed in peripheral blood two months post-transplantation. Blood samples obtained from the tails were treated with red blood cell lysis buffer (eBioscience) and subsequently examined using flow cytometry (BD Accuri C6, BD Biosciences) to ascertain the proportion of GFP^{+} cells. Blood engraftment % is presented in Table S4.

Immunofluorescence, image acquisition and analysis

Murine kidney tissue sections were collected from euthanized mice, fixed using 10% neutral buffered formalin (NBF), and then embedded in paraffin wax. Standard methods were employed to section the tissue at a thickness of 5 µm. After deparaffinization, the sections were transferred to pre-warmed antigen retrieval solution at 95°C for 30 minutes, followed by cooling at room temperature for 20 minutes. Subsequently, the sections were placed in blocking solution (0.25% Triton X-100 and 3% BSA in tris-buffered saline) and then incubated overnight at 4°C with primary antibodies: rabbit/rat anti-NHE3 (1:50; BiCell Scientific) and rabbit anti-GFP (1:500; Abcam). The next day, appropriate Alexa Fluor-conjugated secondary antibodies (Invitrogen) were added for antigen visualization, along with LTL Rhodamine conjugate (1:100; BiCell Scientific) and DAPI stain. Images were captured using a Keyence BZ-X710 digital microscope.

bioRxiv preprint doi: https://doi.org/10.1101/2025.02.12.637793; this version posted February 12, 2025. The copyright holder for this preprint (which was not certified by peer review) is the author/funder, who has granted bioRxiv a license to display the preprint in perpetuity. It is made available under aCC-BY 4.0 International license.

ImagePro Premier software (Media Cybernetics) was utilized for all quantification with thresholding, determining the proportion of expression and colocalization (Pearson's correlation coefficient) for NHE3 and LTL in z-stacks (multiple images taken per section) or stitched images. Regarding Immunocytochemistry (ICC), the cells underwent the following treatments: 4% paraformaldehyde fixation, 0.5% Triton-X-100 permeabilization, and blocking with 2.5% BSA in PBS, following established protocols thereafter.

NHE3-GFP transduced PTCs were seeded at 70% confluence in a 96-well plate with glass-like polymer bottom black frame (P96-1.5P, Cellvis), then fixed with 4% paraformaldehyde (Electron Microscopy Sciences, 15,710,) for 8 min and blocked with 1% BSA (Rockland, BSA-50) in PBS (Corning, 21–031-CV), in the presence of 0.01% saponin (Calbiochem, 558,255), for 1 h. Samples were labeled with the indicated primary antibodies overnight at 4°C in the presence of 0.01% saponin and 1% BSA. Samples were washed 3 times and subsequently incubated with the appropriate combinations of Alexa Fluor (488 or 594)-conjugated anti-goat, or anti-mouse secondary antibodies (Thermo Fisher Scientific, A-32814, A-21203, respectively). Nuclei were stained with Hoechst 33342 (DAPI; Millipore-Sigma, D9542) and samples were preserved in Fluoromount-G reagent (AnaSpec, AS-83218) and kept at 4°C until analyzed. Samples were analyzed with a Zeiss LSM 880 or Zeiss LSM 980 laser-scanning confocal microscope attached to a Axio Observer Z1 microscope at 21°C, using a 63x oil Plan Apo, 1.4-numerical aperture objective. Images were collected using ZEN-LSM software and processed using ImageJ and Adobe Photoshop. The laser power and gain were maintained throughout the experiments to analyze wild-type and *Ctns*$^{-/-}$ cells comparatively. Images were collected and fluorescence intensity and colocalization were quantified using ZEN-LSM software. The following antibodies were used for immunofluorescence in this study: anti-LAMP1 (Santa Cruz Biotechnology, sc-19,992); anti-EEA1 (BD Transduction Laboratories™, 610457); anti-GM130 (BD Transduction Laboratories™, 610822) goat anti-GFP (SICGEN, AB0020-200).

For confocal images, all were acquired using the full dynamic intensity range (1-16383/1-65535) of the specified fluorophores. For quantitative colocalization analysis, regions of interest were drawn around individual cells. Background thresholds were established using secondary antibody controls and further refined using ImageJ's threshold tool to distinguish specific signal from background. These thresholds were typically set between 500-800 (in the 16-bit dynamic range of 0-65536). The weighted colocalization coefficient (Mander's overlap coefficient) was

bioRxiv preprint doi: https://doi.org/10.1101/2025.02.12.637793; this version posted February 12, 2025. The copyright holder for this preprint (which was not certified by peer review) is the author/funder, who has granted bioRxiv a license to display the preprint in perpetuity. It is made available under aCC-BY 4.0 International license.

calculated as $MOC = \sum i(Ri \times Gi)/\sqrt{(\sum iRi^2 \times \sum iGi^2)}$, where Ri and Gi represent the intensity values of corresponding pixels in the red and green channels.
